# Supplementary material for: Splice-Junction-Based Mapping of Alternative Isoforms in the Human Proteome
Source: Cell Rep. Author manuscript; Available in PMC 2020 Jan 15. (PMC6961840; doi:10.1016/j.celrep.2019.11.026)
Supplement: 2 [file NIHMS1546469-supplement-2.zip › DF1/PXD010154/Liver/Liver_26_EEF1A1_RGNVAGDSKNDPPMEAAGFTAQVLVL.pdf]

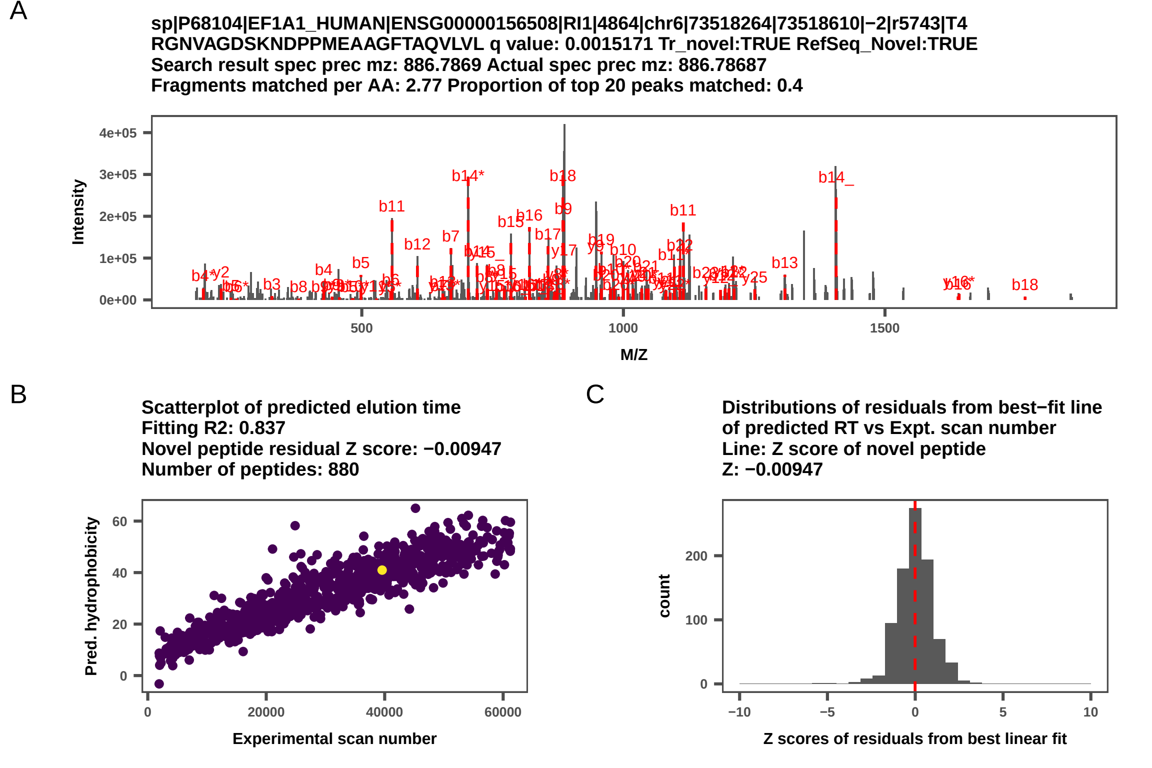

A
sp|P68104|EF1A1_HUMAN|ENSG00000156508|RI1|4864|chr6|73518264|73518610|−2|r5743|T4
RGNVAGDSKNDPPMEAAGFTAQVLVL q value: 0.0015171 Tr_novel:TRUE RefSeq_Novel:TRUE
Search result spec prec mz: 886.7869 Actual spec prec mz: 886.78687
Fragments matched per AA: 2.77 Proportion of top 20 peaks matched: 0.4
Intensity
4e+05
3e+05
2e+05
1e+05
0e+00
500
1000
1500
M/Z
B
Scatterplot of predicted elution time
Fitting R2: 0.837
Novel peptide residual Z score: −0.00947
Number of peptides: 880
Pred. hydrophobicity
60
40
20
0
0
20000
40000
60000
Experimental scan number
C
Distributions of residuals from best−fit line
of predicted RT vs Expt. scan number
Line: Z score of novel peptide
Z: −0.00947
count
200
100
0
−10
−5
0
5
10
Z scores of residuals from best linear fit
